# Routine Vitamin and Iron Biomarkers Do Not Explain Variation in Anti-Müllerian Hormone: Implications for Ovarian Reserve Testing in Clinical Practice

**DOI:** 10.3390/jcm15197571

**Published:** 2026-09-29

**Authors:** Mete Hakan Karalök, Bağnu Dündar, Ayhan Parmaksız, Tugba Elgün, Sevgi Koçyiğit Sevinç, Asiye Gök Yurttaş

**Affiliations:** 1 Department of Obstetrics and Gynecology, Faculty of Medicine, Istanbul Atlas University, Istanbul 34403, Türkiye; hakan.karalok@atlas.edu.tr; 2 Department of Biochemistry, Faculty of Medicine, Istanbul Atlas University, Istanbul 34403, Türkiye; bagnu.dundar@atlas.edu.tr; 3 Department of Biostatistics, Faculty of Medicine, Istanbul Health and Technology University, Istanbul 34445, Türkiye; ayhan.parmaksiz@istun.edu.tr; 4 Department of Medical Biology, Faculty of Medicine, Biruni University, Istanbul 34015, Türkiye; telgun@biruni.edu.tr; 5 Biruni University Research Center (B@MER), Biruni University, Istanbul 34015, Türkiye; 6 Department of Biophysics, Faculty of Medicine, Kutahya Health Sciences University, Kutahya 43100, Türkiye; sevgi.kocyigitsevinc@ksbu.edu.tr

**Keywords:** anti-Müllerian hormone, ovarian reserve, vitamin D, vitamin B12, ferritin, iron metabolism

## Abstract

**Background:** Anti-Müllerian hormone (AMH) is a key biomarker of ovarian reserve, reflecting the quantity of the remaining follicular pool. While chronological age is the primary determinant of AMH concentrations, emerging evidence suggests that vitamin D, vitamin B12, folate, and iron metabolism parameters may also influence ovarian function through mechanisms involving steroidogenesis, DNA synthesis, and oxidative stress regulation. However, clinical findings regarding these associations remain inconsistent. **Objective:** The aim of this study was to evaluate the relationships between serum AMH concentrations and biomarkers of vitamin D status, vitamin B12, folate, ferritin, and lipid profile in women of reproductive age, and to compare the strength of these associations with that of chronological age. **Methods:** This retrospective, observational, cross-sectional study included 264 women who underwent AMH testing at Atlas University Hospital. Demographic and laboratory data including AMH, age, vitamin D, vitamin B12, folic acid, ferritin, and lipid parameters were extracted from electronic medical records. Bivariate correlations were assessed using Pearson’s and Spearman’s coefficients on both untransformed and Box–Cox power-transformed data, and were repeated as first-order partial correlations conditioning on chronological age. Statistical significance was set at *p* < 0.05. **Results:** The mean age was 34.61 ± 5.75 years, and median AMH was 0.96 ng/mL (Q1–Q3: 0.34–2.34). Age showed a moderate-to-strong, significant inverse correlation with AMH (Pearson’s r = −0.508, Spearman’s rho = −0.547; *p* < 0.001), and the linear component of this association strengthened after Box–Cox transformation (Pearson’s r = −0.553; *p* < 0.001). In contrast, vitamin D, vitamin B12, folic acid, ferritin, and lipid parameters (triglycerides, HDL, LDL, total cholesterol) showed no statistically significant and no clinically meaningful correlation with AMH, with all coefficients remaining below 0.20 regardless of transformation (all *p* > 0.05). After adjustment for age, every coefficient attenuated further towards the null (all |r_partial_| < 0.10; all *p* ≥ 0.127), the largest being that of 25-hydroxyvitamin D (r_partial_ = 0.094, 95% CI −0.027 to 0.213). A sensitivity power analysis confirmed 80% power to detect an age-adjusted partial correlation of |r| = 0.172 and 91% power at |r| = 0.200. **Conclusions:** Age remains the principal determinant of ovarian reserve, while vitamin D, vitamin B12, folic acid, ferritin, and lipid parameters show no statistically significant or clinically meaningful association with AMH. These findings suggest that any biological influence of these micronutrients on ovarian reserve, if present, is markedly overshadowed by age-dependent follicular decline. Prospective studies incorporating longitudinal AMH measurements and standardized nutritional assessment are needed to further clarify these relationships.

## 1. Introduction

Ovarian reserve, defined as the quantity and developmental competence of the remaining follicular pool, constitutes a central determinant of female reproductive potential. Among the available biomarkers used to evaluate ovarian reserve, anti-Müllerian hormone (AMH) is considered one of the most reliable indicators because of its minimal intra-cycle variability and strong correlation with the population of growing ovarian follicles. Produced by granulosa cells of preantral and small antral follicles, AMH reflects functional follicular activity and has become an indispensable tool in reproductive medicine for assessing fertility potential, predicting ovarian response during assisted reproductive technologies, and evaluating reproductive aging [1,2,3,4].

Although chronological age remains the most important determinant of AMH concentrations and ovarian reserve decline, growing evidence suggests that ovarian function may also be influenced by various metabolic, endocrine, nutritional, and environmental factors. Ovarian folliculogenesis is a highly regulated biological process that depends on adequate cellular proliferation, mitochondrial activity, steroidogenesis, DNA synthesis, epigenetic regulation, and protection against oxidative stress. Consequently, alterations in micronutrient status and iron homeostasis may potentially affect ovarian physiology and reproductive outcomes [5,6].

Rather than affecting “ovarian function” broadly, the biological plausibility for an association with AMH specifically rests on mechanisms acting at the level of the granulosa cells of preantral and small antral follicles—the exclusive source of circulating AMH. For vitamin D, this is supported by the presence of a vitamin D response element (VDRE) directly within the AMH gene promoter [7], providing a direct transcriptional mechanism rather than a generic metabolic effect. For vitamin B12 and folate, the plausible pathway is through one-carbon metabolism and DNA methylation: enzymes of the methionine–folate cycle are expressed in granulosa cells, and adequate flux through this pathway supports the S-adenosylmethionine-dependent methylation changes that accompany granulosa cell proliferation during follicular recruitment and growth [8]; deficiency could theoretically impair this process and reduce the pool of actively growing, AMH-producing follicles. For ferritin/iron status, the mechanism relates to iron’s role in granulosa cell mitochondrial and redox metabolism: granulosa cells are particularly susceptible to iron-related oxidative injury due to their high mitochondrial density and active steroidogenic machinery, and both iron deficiency and iron overload—the latter through Fenton-reaction-driven reactive oxygen species and ferroptotic lipid peroxidation—have been linked to granulosa cell apoptosis and follicular atresia, potentially depleting the AMH-secreting follicle pool [9]. For lipid parameters, cholesterol serves as the obligate precursor for steroid hormone synthesis in granulosa and theca cells, which take up circulating lipoprotein-derived cholesterol as steroidogenic substrate, providing a theoretical, though less direct, link to follicular microenvironment health [10].

Among these factors, vitamin D has attracted considerable attention because vitamin D receptors (VDR) and vitamin D-metabolizing enzymes are expressed throughout the female reproductive tract, including the ovary, endometrium, and placenta. Experimental studies have demonstrated that vitamin D may modulate follicular development, steroid hormone synthesis, granulosa cell function, and reproductive endocrine signaling pathways. These observations have led to the hypothesis that vitamin D deficiency could contribute to diminished ovarian reserve and impaired fertility. However, clinical investigations evaluating the relationship between vitamin D status and AMH concentrations have yielded inconsistent findings. While some studies have reported positive associations between serum vitamin D and AMH levels, others have failed to demonstrate significant correlations, highlighting the need for further investigation [11,12,13,14].

Vitamin B12 and folate are also essential micronutrients involved in one-carbon metabolism, DNA synthesis, methylation reactions, and cellular replication. Adequate availability of these vitamins is critical for oocyte maturation, embryonic development, and maintenance of genomic stability. Deficiencies may impair cell division and reproductive function through disruptions in methylation-dependent regulatory mechanisms and increased homocysteine accumulation. Nevertheless, despite their recognized importance in reproductive biology, the relationship between vitamin B12, folate status, and ovarian reserve markers remains insufficiently explored, and available evidence is limited [15,16].

Iron metabolism may represent another important but underinvestigated determinant of ovarian function. Ferritin, the principal intracellular iron-storage protein, is widely used as a biomarker of body iron reserves. Both iron deficiency and iron overload can disrupt cellular homeostasis through oxidative stress generation, mitochondrial dysfunction, and impaired energy metabolism. Since ovarian follicles are highly metabolically active structures, disturbances in iron balance may adversely affect follicular growth, oocyte quality, and reproductive capacity. Although emerging studies suggest potential associations between iron-related biomarkers and reproductive outcomes, the relationship between ferritin, other iron metabolism parameters, and AMH concentrations remains poorly characterized [17,18].

Despite increasing interest in the interaction between nutritional status and ovarian reserve, the current literature remains fragmented and sometimes contradictory. Most previous studies have focused on single biomarkers, involved relatively small sample sizes, or were conducted in selected populations such as infertile women or patients undergoing assisted reproductive treatment. Comprehensive evaluations simultaneously examining vitamin D, vitamin B12, folate, ferritin, and additional iron metabolism parameters in reproductive-aged women are relatively scarce [11,12,13,14,15,16,17,18].

Given these limitations, further research is warranted to clarify whether vitamin and iron metabolism biomarkers are associated with ovarian reserve independently of age and other potential confounding factors. Improved understanding of these relationships may contribute to a more comprehensive interpretation of ovarian reserve assessment and facilitate the identification of potentially modifiable biological factors involved in female reproductive health.

Although several studies have evaluated the relationship between AMH and vitamin D, findings remain inconsistent, and most previous studies focused on infertility, PCOS, or IVF populations. Data from routine laboratory populations across a broad reproductive age range are scarce. Furthermore, few studies have simultaneously evaluated vitamin B12, folate, ferritin, and vitamin D together with AMH concentrations across a wide reproductive age range. Therefore, the present study aimed to retrospectively investigate the associations between serum AMH concentrations and vitamin D, vitamin B12, folate, ferritin, and lipid parameters in women of reproductive age. Furthermore, we sought to compare the strength of these associations with that of chronological age, in order to establish whether any of these biochemical markers warrants further multivariable investigation.

## 2. Material and Methods

### 2.1. Study Design and Objective

This retrospective, observational, cross-sectional study was conducted to investigate the associations between serum anti-Müllerian hormone (AMH) concentrations and biomarkers of vitamin and iron metabolism in women of reproductive age. Specifically, the study aimed to evaluate the relationship between AMH levels and serum vitamin D, vitamin B12, folate, ferritin, and lipid parameters, and to compare the strength of these associations with that of chronological age.

### 2.2. Study Setting and Population

**Figure 1:**
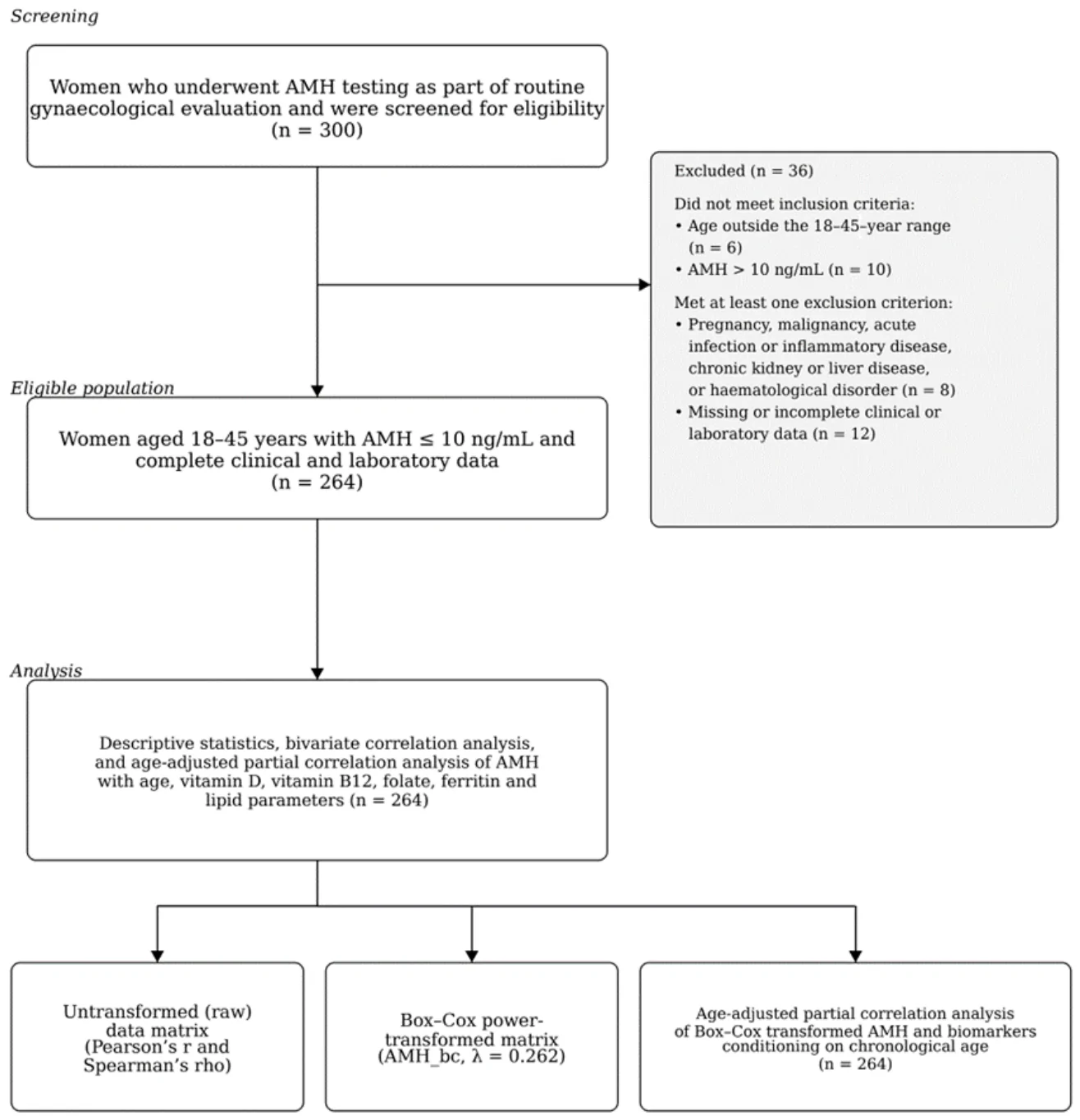
Flow diagram of patient selection. Of the 300 women screened, 36 were excluded because they did not meet the inclusion criteria or met at least one exclusion criterion, leaving 264 women in the final analysis. AMH, anti-Müllerian hormone.

The study was carried out at Atlas University Hospital, a tertiary care academic medical center. Electronic medical records of women who attended the Department of Obstetrics and Gynecology and underwent AMH testing as part of routine clinical evaluation were retrospectively reviewed. The study population consisted of women whose laboratory assessments included measurements of AMH and relevant vitamin, iron-status, and lipid parameters. Of the initial 300 patients screened, 36 did not meet the eligibility criteria and were excluded: patients whose age fell outside the 18–45-year range, patients with serum AMH concentrations exceeding 10 ng/mL according to the predefined analytic eligibility threshold, patients meeting at least one of the predefined clinical exclusion criteria, and patients with missing or incomplete clinical or laboratory results for one or more of the study variables. Consequently, the final statistical analyses were conducted on 264 patients. The patient selection process is summarised in Figure 1.

### 2.3. Eligibility Criteria

#### 2.3.1. Inclusion Criteria

Women were eligible for inclusion if they met all of the following criteria:

- Female patients aged 18–45 years at the time of laboratory evaluation;
- Female patients with AMH testing performed as part of routine gynecological evaluation;
- Availability of demographic data, including age;
- Availability of serum AMH measurement;
- Serum AMH concentration ≤ 10 ng/mL.

A further qualification of this eligibility rule requires explicit statement. A threshold applied to AMH alone is an imperfect screen for polycystic ovary syndrome. It will not identify women who satisfy the Rotterdam criteria on the basis of oligo-anovulation and clinical or biochemical hyperandrogenism while their AMH lies within the ordinary range, and it does not distinguish polycystic ovarian morphology from the syndrome itself. This matters for the present analysis because polycystic ovary syndrome is associated with alterations of iron status and of vitamin D status, that is, with precisely the biomarkers examined here, so that its presence in the cohort is a potential source of confounding rather than a neutral admixture. A separate exclusion based on a recorded diagnosis of polycystic ovary syndrome was therefore considered. The direction of the residual bias is also worth stating: because women with the syndrome tend to have both higher AMH and altered vitamin D and ferritin concentrations, their retention in a cohort would be expected to generate a spurious association rather than to conceal a true one, so residual contamination of this kind is an unlikely explanation for the null findings reported here. The point is returned to among the limitations.

- Availability of laboratory results for vitamin D, vitamin B12, folic acid, ferritin, and lipid parameters;
- Complete demographic and laboratory data required for the final analysis.

#### 2.3.2. Exclusion Criteria

Participants were excluded if any of the following conditions were present:

- Pregnancy at the time of laboratory evaluation;
- History of malignancy;
- Acute infection or active inflammatory disease;
- Chronic kidney disease or chronic liver disease;
- History of hematological disorders;
- Missing or incomplete clinical or laboratory data.

### 2.4. Data Collection

Data were retrospectively extracted from the Hospital Information Management System (HIMS) and Laboratory Information Management System (LIMS) of Atlas University Hospital. Data extraction from HIMS and LIMS was performed independently by two investigators (MHK and TE) using a standardized data collection form; discrepancies were resolved by consensus with a third investigator (BD). The following variables were collected: age, serum AMH concentration, 25-hydroxyvitamin D, vitamin B12, folate, ferritin, triglycerides, total cholesterol, HDL cholesterol and LDL cholesterol. Ferritin was used as the marker of iron status, since it was the only iron-related parameter available for the entire study population. All laboratory measurements had been performed as part of standard clinical practice in the hospital’s central laboratory. No additional blood samples were obtained specifically for the purposes of this study. Because the study was based exclusively on existing electronic medical records, no questionnaires, interviews, psychometric assessments, or patient-reported outcome measures were utilized.

### 2.5. Laboratory Measurements

All laboratory measurements were performed as part of routine clinical care in the central laboratory of Atlas University Hospital, in accordance with the respective manufacturers’ instructions and the laboratory’s standard operating procedures. Venous blood was collected into plain tubes without anticoagulant for all biochemical and immunoassay determinations. All parameters were analysed on the day of collection, and no sample underwent freeze–thaw cycling prior to analysis.

Serum AMH concentrations were measured using the VIDAS^®^ AMH assay (Ref. 417011; bioMérieux, Marcy-l’Étoile, France) on the VIDAS^®^ 3 automated immunoassay platform, based on the enzyme-linked fluorescent assay (ELFA) principle. The assay is validated for the quantitative determination of AMH in human serum and lithium-heparin plasma; serum was used in the present study, with a required sample volume of 200 μL. According to the manufacturer’s specifications, the validated analytical measurement range is 0.02–9.00 ng/mL, the limit of detection (LoD) is 0.01 ng/mL and the lower limit of quantification (LoQ) is 0.02 ng/mL. Samples with concentrations exceeding 9.00 ng/mL were diluted according to the manufacturer’s recommended procedure and reanalysed, with the final concentration calculated using the appropriate dilution factor. In the manufacturer’s precision evaluation, performed at five concentration levels (0.22, 1.08, 2.99, 5.45 and 7.37 ng/mL), coefficients of variation (CVs) for repeatability (within-run precision) ranged from 4.1% to 4.8%, intra-lot CVs from 6.6% to 8.2% and intra-laboratory precision CVs from 8.3% to 10.6%; the CV between reagent lots did not exceed 11%. Calibration and internal quality control were performed according to the manufacturer’s instructions using the S1 calibrator and C1 control, with recalibration following reagent-lot changes. The same VIDAS^®^ AMH assay and VIDAS^®^ 3 platform were used throughout the study period, with no change in assay platform or reagent generation.

Serum 25-hydroxyvitamin D, vitamin B12, folate and ferritin were measured by chemiluminescent microparticle immunoassay (CMIA) on an automated immunoassay analyser (ARCHITECT i2000, Abbott Laboratories, Abbott Park, IL, USA) using Abbott Diagnostics reagent kits and the manufacturer’s validated assay protocols. Because vitamin D concentrations are subject to seasonal variation, samples were not stratified by collection season; this is addressed further as a study limitation.

Triglycerides, total cholesterol, high-density lipoprotein (HDL) cholesterol and low-density lipoprotein (LDL) cholesterol were measured on an automated clinical chemistry analyser (ARCHITECT c8000, Abbott Laboratories, Abbott Park, IL, USA) using Abbott Diagnostics reagent kits and the manufacturer’s validated assay protocols, by enzymatic colorimetric methods.

Internal quality control was performed daily at two concentration levels for each analyte, and the laboratory participated in an external quality assessment scheme throughout the study period. Patient results were released only when internal quality-control results met the laboratory’s predefined acceptance criteria. The same analytical platforms and reagent systems were used throughout the study period; no change of platform, reagent generation or reporting limits occurred, and no cross-platform harmonisation was therefore required.

### 2.6. Statistical Analysis

Continuous variables were summarized using descriptive statistics, including mean ± standard deviation (SD), median with interquartile range (Q1–Q3), and minimum–maximum values. Bivariate relationship structures between AMH and clinical, metabolic, lipid, vitamin, and iron-related parameters were evaluated in two phases: first using the untransformed raw data matrix and subsequently using Box–Cox power-transformed variables to assess whether linear relationships improved after distributional adjustment. Pearson’s correlation coefficient (r) was used to quantify linear associations, while Spearman’s rank correlation coefficient (rho) was used to evaluate monotonic associations. Statistical significance was set at a two-tailed *p*-value < 0.05. All computational pipelines and statistical analyses were performed using R software (version 4.5.1; R Foundation for Statistical Computing, Vienna, Austria), including the Hmisc (v5.2.4), MASS (v7.3.65), and dplyr (v1.1.4) packages. Because chronological age is the dominant determinant of AMH, and because a null unadjusted coefficient does not by itself exclude an association that emerges only once age has been accounted for, the bivariate analysis was followed by a first-order partial correlation analysis conditioning on age. Partial correlations were computed between Box–Cox-transformed AMH and each Box–Cox-transformed biomarker, with chronological age as the single conditioning covariate, and 95% confidence intervals were obtained by the Fisher z transformation with df = n − 3 = 261. The optimal transformation parameters for every variable, together with the raw and transformed distributional characteristics, are reported in Supplementary Table S1. A first-order partial correlation of this kind is algebraically equivalent to the standardized regression coefficient of the corresponding biomarker in a linear model containing age and that biomarker, so the age-adjusted estimate requested for each predictor is reported in full in the Results. A formal a priori power calculation was not performed, as this was a retrospective analysis of all eligible records available within the study period; a sensitivity analysis was therefore carried out to establish what the achieved sample size can and cannot detect, and it was extended to the adjusted analyses because conditioning on chronological age is the situation in which power is most commonly lost. With n = 264, a two-tailed α of 0.05 and 80% power, the smallest detectable bivariate correlation is |r| = 0.17. For a first-order partial correlation controlling for age, which is the analysis actually reported, the smallest detectable coefficient is |r| = 0.172, corresponding to a Cohen f^2^ of 0.030; the same sample confers 91.0% power at |r| = 0.200 and 68.8% at |r| = 0.150 (Table 1). The full sensitivity curve across hypothesized partial correlations is given in Supplementary Table S2. Because a partial correlation below 0.200 accounts for less than 4% of the residual variance in serum AMH, effects smaller than this were treated as a pragmatic threshold of limited clinical relevance for interpretation. Extending the calculation to a fully specified linear model containing age and all eight biomarkers simultaneously, the smallest effect of a single predictor detectable at 80% power corresponds to a semi-partial correlation of 0.171, equivalently a Cohen f^2^ of 0.030 or an increment in R^2^ of 0.029; this figure is invariant to three decimal places across models containing between two and twelve predictors, because at this sample size the residual degrees of freedom remain large relative to the number of estimated parameters. Adjustment for age therefore does not materially reduce the detectable effect in this dataset, and the negative findings reported below cannot be attributed to a loss of power on adjustment. The precision of the null result can be stated directly rather than inferred: at n = 264 the 95% confidence interval around an observed correlation of zero extends from −0.12 to +0.12, so these data exclude, with 95% confidence, any linear association stronger than |r| ≈ 0.12 between AMH and the biomarkers examined, and the confidence intervals reported in Table 2, Table 3 and Table 4 allow the precision of the bivariate and age-adjusted estimates to be read separately for every coefficient. Given the exploratory nature of this study and the fact that nine bivariate correlations were tested against a single primary outcome (AMH), a formal correction for multiple comparisons (e.g., Bonferroni) was not applied, as such corrections increase the risk of type II error and were considered overly conservative for a hypothesis-generating analysis; however, we note that even without correction, none of the non-age biomarkers approached conventional statistical significance (all *p* > 0.05), and effect sizes remained negligible (|r| and |ρ| < 0.20) regardless of the multiple testing framework applied. Subgroup analyses stratifying the cohort by age category or by vitamin D deficiency/insufficiency status were not performed in the primary analysis, as the sample size within resulting subgroups would have limited statistical power; however, we agree that such analyses could provide additional insight and have noted this as a direction for future research with larger, prospectively designed cohorts.

### 2.7. Ethical Considerations

The study protocol was approved by the Istanbul Atlas University Non-Interventional Clinical Research Ethics Committee (Approval No. 06/07, dated 22 June 2026) prior to data collection. Permission to access hospital records was obtained from the relevant institutional authorities. All data were anonymized before analysis, and no personally identifiable information was recorded. As the study was retrospective and utilized routinely collected clinical data, direct patient contact was not required. The study was conducted in accordance with the ethical principles outlined in the Declaration of Helsinki and its subsequent amendments.

## 3. Results

### 3.1. Study Population and Descriptive Characteristics

Out of the initial sample of 300 patients screened, 36 were excluded from the study population because they did not meet the age or AMH inclusion criteria, met at least one predefined clinical exclusion criterion, or had missing or incomplete clinical or laboratory data; the corresponding numbers for each reason are shown in Figure 1. Consequently, the final descriptive and statistical evaluations were conducted on 264 patients. The clinical and demographic characteristics of the study cohort are comprehensively summarized in Table 1.

The mean age of the participants was 34.61 ± 5.75 years, ranging from 19.20 to 44.83 years. In the final study population, serum AMH levels demonstrated a mean value of 1.58 ± 1.71 ng/mL, with a median value of 0.96 ng/mL (Q1–Q3: 0.34–2.34 ng/mL) and a range of 0.02 to 9.60 ng/mL.

Regarding the metabolic and lipid profiles, the study population exhibited a mean total cholesterol of 189.03 ± 35.58 mg/dL, a mean LDL cholesterol of 112.38 ± 27.64 mg/dL, and a mean HDL cholesterol of 57.90 ± 13.83 mg/dL. Triglyceride levels showed a wide distribution range (26.00–686.00 mg/dL), with a median concentration of 77.00 mg/dL. The micronutrient status of the cohort revealed median concentrations of 22.75 ng/mL for ferritin, 330.00 pg/mL for vitamin B12, 6.85 ng/mL for folic acid, and 21.70 ng/mL for 25-hydroxyvitamin D.

**Table 1:** Descriptive Characteristics of the Study Population (n = 264).

Variable	Mean ± SD	Median (Q1–Q3)	Min–Max
AMH (ng/mL)	1.58 ± 1.71	0.96 (0.34–2.34)	0.02–9.60
Age (Years)	34.61 ± 5.75	35.14 (29.91–38.99)	19.20–44.83
TG (mg/dL)	93.99 ± 78.61	77.00 (55.00–99.00)	26.00–686.00
HDL (mg/dL)	57.90 ± 13.83	58.00 (48.68–66.12)	28.50–101.10
LDL (mg/dL)	112.38 ± 27.64	109.35 (91.00–132.25)	53.00–186.00
TC (mg/dL)	189.03 ± 35.58	184.00 (164.00–213.25)	106.00–332.00
Ferritin (ng/mL)	36.24 ± 43.98	22.75 (11.20–40.67)	1.00–370.11
Vit-B12 (pg/mL)	371.97 ± 195.53	330.00 (252.75–430.50)	94.00–1952.00
Folic Acid (ng/mL)	7.40 ± 3.54	6.85 (4.88–8.80)	2.00–32.00
Vit-D 25OH (ng/mL)	23.22 ± 14.88	21.70 (13.10–28.97)	3.50–136.80

Note: AMH: Anti-Müllerian Hormone, TG: Triglycerides, HDL: High-Density Lipoprotein, LDL: Low-Density Lipoprotein, TC: Total Cholesterol, Vit-B12: Vitamin B12, Vit-D 25OH: 25-Hydroxyvitamin D.

### 3.2. Bivariate Correlation Analysis

To evaluate potential links between serum AMH levels and clinical, metabolic, lipid, vitamin, and iron-related parameters, bivariate correlation structures were analyzed and compared across both untransformed raw (Table 2) and Box–Cox power-transformed data matrices (Table 3).

In the raw data analysis, age demonstrated a statistically significant moderate-to-strong negative correlation with untransformed AMH levels (Pearson’s r = −0.508, Spearman’s rho = −0.547; *p* < 0.001). Among the remaining raw lipid and biochemical markers, none reached formal statistical significance under either the linear or the monotonic framework. The values closest to the conventional threshold were observed for HDL cholesterol (Pearson’s r = −0.118, *p* = 0.055) and ferritin (Pearson’s r = 0.118, *p* = 0.055); both remained non-significant and negligible in magnitude, and all other coefficients were accompanied by *p*-values above 0.10.

**Table 2:** Correlation Matrices for Untransformed (Raw) AMH (n = 264).

Variable	Pearson’s r (95% CI)	*p*	Spearman’s rho (95% CI)	*p*
Age	−0.508 (−0.592, −0.413)	<0.001	−0.547 (−0.631, −0.449)	<0.001
TG	0.085 (−0.036, 0.203)	0.17	−0.098 (−0.216, 0.024)	0.114
HDL	−0.118 (−0.236, 0.003)	0.055	−0.092 (−0.211, 0.029)	0.136
LDL	−0.007 (−0.127, 0.114)	0.913	−0.045 (−0.165, 0.076)	0.465
TC	−0.025 (−0.145, 0.096)	0.684	−0.067 (−0.187, 0.054)	0.276
Ferritin	0.118 (−0.002, 0.236)	0.055	0.023 (−0.098, 0.144)	0.706
Vit-B12	−0.027 (−0.147, 0.094)	0.666	0.048 (−0.073, 0.168)	0.436
Folic Acid	−0.059 (−0.179, 0.062)	0.338	−0.039 (−0.159, 0.083)	0.533
Vit-D 25OH	−0.018 (−0.139, 0.103)	0.769	0.063 (−0.058, 0.182)	0.309

Note: AMH: Anti-Müllerian Hormone, TG: Triglycerides, HDL: High-Density Lipoprotein, LDL: Low-Density Lipoprotein, TC: Total Cholesterol, Vit_B12: Vitamin B12, Vit-D 25OH: 25-Hydroxyvitamin D. CI: Confidence Interval. Pearson correlation confidence intervals were derived using the Fisher z-transformation; Spearman rank correlation confidence intervals were derived using the Bonett–Wright standard error.

Following Box–Cox power transformation, the linear association between age and transformed AMH was strengthened (Pearson’s r = −0.553, *p* < 0.001), while the rank-based Spearman correlation remained unchanged (rho = −0.547, *p* < 0.001), indicating that transformation improved the linear representation of the age–AMH relationship. In contrast, the transformations did not produce clinically meaningful improvements for any of the metabolic, lipid, vitamin, or iron-related parameters. TG_bc showed no significant association with AMH_bc under either framework (Pearson’s r = −0.085, *p* = 0.170; Spearman’s rho = −0.098, *p* = 0.114), and all other transformed variables—HDL_bc, LDL_bc, TC_bc, Ferritin_bc, Vit-B12_bc, Folic Acid_bc, and Vit-D 25OH_bc—likewise failed to demonstrate any substantial correlation with AMH_bc (*p* > 0.05 for all Pearson and Spearman coefficients). All non-age parameters showed correlation coefficients below 0.20, indicating negligible effect sizes. These unadjusted coefficients are examined again after adjustment for chronological age in the following section.

**Figure 2:**
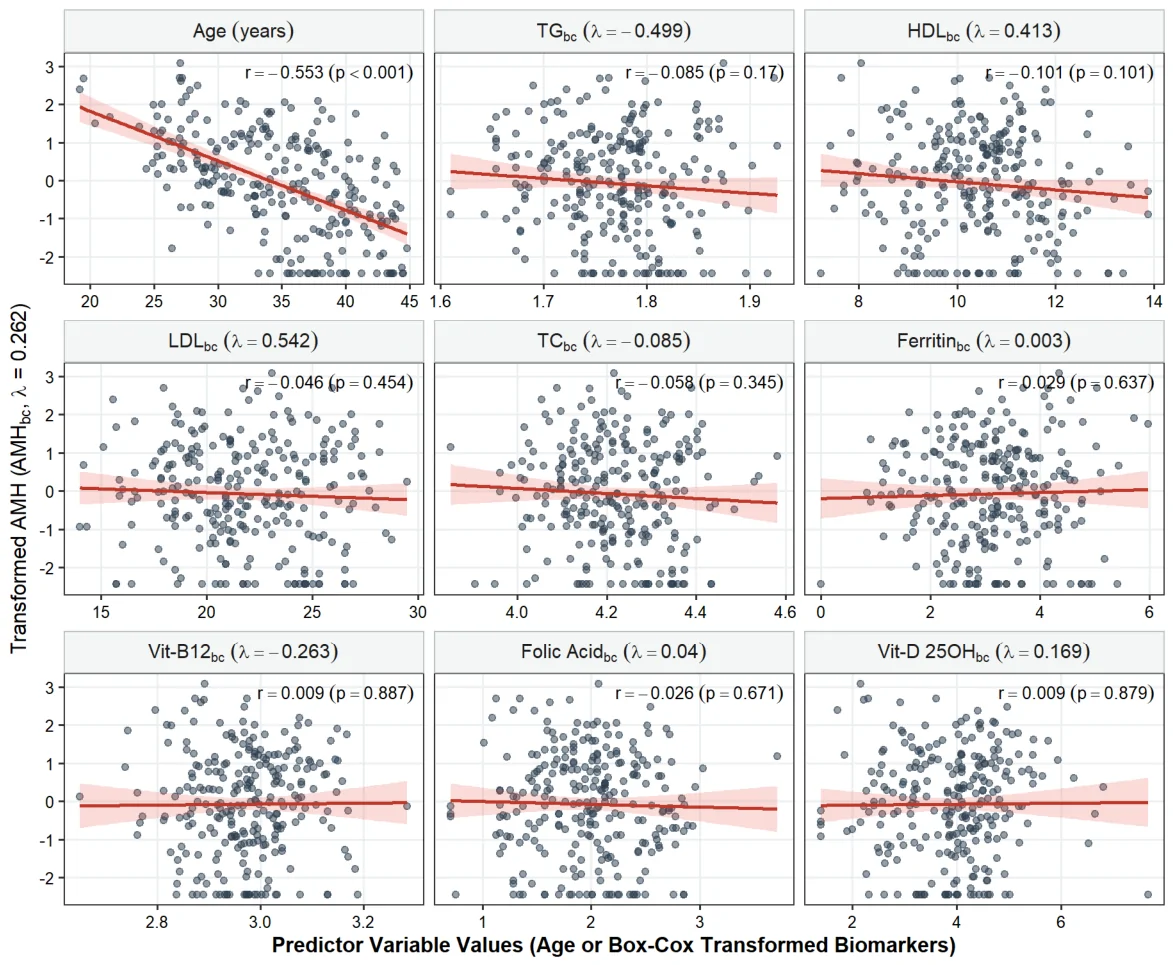
Scatter plots displaying the bivariate relationships between transformed anti-Müllerian hormone (AMH_bc) and chronological age alongside Box–Cox transformed biochemical predictors (n = 264).

The bivariate relationships are displayed graphically in Figure 2, which presents scatterplots of Box–Cox-transformed AMH (AMH_bc) against chronological age and against each of the transformed vitamin, iron-status and lipid parameters, with the fitted linear trend superimposed on each panel. The contrast between the age panel and the remaining panels reproduces visually what the coefficients of Table 2 and Table 3 report numerically.

**Table 3:** Correlation Matrices for Box–Cox-Transformed AMH (AMH_bc).

Variable	Pearson’s r	*p*	Spearman’s rho	*p*
Age	−0.553 (−0.632, −0.463)	<0.001	−0.547 (−0.631, −0.449)	<0.001
TG_bc (λ = −0.499)	−0.085 (−0.203, 0.036)	0.17	−0.098 (−0.216, 0.024)	0.114
HDL_bc (λ = 0.413)	−0.101 (−0.219, 0.020)	0.101	−0.092 (−0.211, 0.029)	0.136
LDL_bc (λ = 0.542)	−0.046 (−0.166, 0.075)	0.454	−0.045 (−0.165, 0.076)	0.465
TC_bc (λ = −0.085)	−0.058 (−0.178, 0.063)	0.345	−0.067 (−0.187, 0.054)	0.276
Ferritin_bc (λ = 0.003)	0.029 (−0.092, 0.149)	0.637	0.023 (−0.098, 0.144)	0.706
Vit-B12_bc (λ = −0.263)	0.009 (−0.112, 0.129)	0.887	0.048 (−0.073, 0.168)	0.436
Folic Acid_bc (λ = 0.040)	−0.026 (−0.147, 0.095)	0.671	−0.039 (−0.159, 0.083)	0.533
Vit-D 25OH_bc (λ = 0.169)	0.009 (−0.111, 0.130)	0.879	0.063 (−0.058, 0.182)	0.309

Note: The subscript “_bc” denotes variables subjected to Box–Cox power transformation. Age remained untransformed. AMH_bc was transformed using optimal λ = 0.262. Pearson correlation confidence intervals were derived using the Fisher z-transformation; Spearman rank correlation confidence intervals were derived using the Bonett–Wright standard error (n = 264). AMH: Anti-Müllerian hormone; TG: triglycerides; HDL: high-density lipoprotein; LDL: low-density lipoprotein; TC: total cholesterol; Vit-D 25OH: 25-hydroxyvitamin D.

Crucially, none of the clinical, metabolic, lipid, vitamin, or iron-related markers achieved formal statistical significance in either the raw or the Box–Cox-transformed matrix, and the absolute magnitude of all observed correlation coefficients for these markers remained consistently below the 0.20 threshold (r < 0.20 and rho < 0.20). Coefficients of this magnitude indicate an extremely weak relationship and a negligible effect size, implying that these predictors share only a trivial fraction of common variance with AMH. These bivariate coefficients are nevertheless unadjusted, and a weak marginal association does not by itself exclude an association that emerges only after chronological age has been accounted for. The age-adjusted partial correlation analysis specified in the Section 2.6 was therefore performed to address that possibility directly, and its results are reported below.

Age-Adjusted Partial Correlation Analysis

To isolate the association of each circulating micronutrient and metabolic marker with ovarian reserve independently of chronological age, first-order partial correlations were computed between Box–Cox-transformed AMH and each transformed biomarker, conditioning on age (Table 4). Every coefficient attenuated toward the null after adjustment, with all |r_partial_| below 0.10, and none approached statistical significance (all *p* ≥ 0.127). The largest coefficient was that of 25-hydroxyvitamin D (r_partial_ = 0.094, 95% CI −0.027 to 0.213, *p* = 0.127), which accounts for less than 1% of the residual variance in transformed AMH; the remaining coefficients ranged from −0.049 for HDL cholesterol to 0.060 for vitamin B12. The confidence intervals are informative in themselves: the widest of them extends only to 0.213, so that even the upper bound of the most favourable estimate remains below the pragmatic interpretive threshold of 0.200 used to denote limited clinical relevance.

These adjusted estimates are consistent in direction and magnitude with the unadjusted coefficients of Table 2 and Table 3, and they answer the question that the bivariate analysis alone could not: the absence of an association is not an artefact of the dominant age effect, since it persists once age is removed from both variables. Because each first-order partial correlation is the standardized coefficient that the corresponding biomarker would take in a linear model containing age and that biomarker, the age-adjusted estimate for every predictor is reported here in full. A single model entering all nine predictors simultaneously was not additionally fitted. Two considerations argued against it: none of the eight biomarkers retains an association after adjustment for age, so no coefficient would be altered by mutual adjustment in a manner that could change the conclusion; and the four lipid fractions are structurally collinear, since total cholesterol is close to a linear function of HDL, LDL and triglycerides, so that entering them together would inflate the standard errors of individually null estimates without adding information. The sensitivity analysis reported in the Section 2.6 shows that such a model would in any case have retained 80% power to detect a semi-partial correlation of 0.171, and no effect approaching that magnitude was observed.

**Table 4:** First-order partial correlation coefficients between Box–Cox-transformed serum AMH (AMH_bc) and Box–Cox-transformed metabolic and micronutrient biomarkers after adjustment for chronological age (n = 264).

Variable	Age-Adjusted Partial r	95% CI	*p*
TG_bc (λ = −0.499)	−0.040	−0.160 to 0.082	0.523
HDL_bc (λ = 0.413)	−0.049	−0.169 to 0.072	0.426
LDL_bc (λ = 0.542)	0.029	−0.092 to 0.150	0.637
TC_bc (λ = −0.085)	0.042	−0.080 to 0.162	0.500
Ferritin_bc (λ = 0.003)	0.055	−0.067 to 0.174	0.378
Vit-B12_bc (λ = −0.263)	0.060	−0.061 to 0.180	0.333
Folic Acid_bc (λ = 0.040)	0.009	−0.113 to 0.129	0.890
Vit-D 25OH_bc (λ = 0.169)	0.094	−0.027 to 0.213	0.127

Note: Both AMH and the biomarker variables were normalized by optimal maximum-likelihood Box–Cox power transformation, with the optimal λ given in parentheses and reported in full in Supplementary Table S1. Chronological age served as the single conditioning covariate (k = 1). Confidence intervals were computed using the Fisher z transformation with df = n − 3 = 261. TG: triglycerides; HDL: high-density lipoprotein cholesterol; LDL: low-density lipoprotein cholesterol; TC: total cholesterol; Vit-B12: vitamin B12; Vit-D 25OH: 25-hydroxyvitamin D.

Vitamin D Status Distribution

Because the absence of an association with vitamin D is easier to interpret when the distribution of vitamin D status in the cohort is known, participants were additionally classified using the conventional clinical thresholds of deficiency (25-hydroxyvitamin D below 20 ng/mL), insufficiency (20 to 29 ng/mL) and sufficiency (30 ng/mL or above). Vitamin D status was deficient in 113 women (42.8%), insufficient in 90 (34.1%) and sufficient in 61 (23.1%) (Table 5). This distribution is relevant to the interpretation of the null result, since a cohort concentrated within a narrow band of vitamin D status would restrict the range over which an association could be detected; the observed spread is reported here so that readers can judge whether restriction of range contributes to the absence of a correlation.

## 4. Discussion

The present study investigated the association between serum anti-Müllerian hormone (AMH) concentrations and biomarkers of vitamin status and iron metabolism in women of reproductive age. AMH is widely recognized as a reliable marker of ovarian reserve, reflecting the quantity of small growing follicles and serving as an important predictor of reproductive potential. While age remains the dominant determinant of AMH levels, increasing attention has been directed toward potentially modifiable factors that may influence ovarian function. In this context, the current study sought to clarify whether vitamin D, vitamin B12, folate and ferritin are associated with ovarian reserve, and how the strength of any such association compares with that of chronological age.

Consistent with previous evidence, age demonstrated a significant inverse relationship with AMH levels. The age-related decline in AMH is well established and reflects the progressive depletion of the ovarian follicular pool throughout reproductive life [2,3]. Therefore, age must be taken into account when interpreting any apparent association between AMH and biochemical parameters. In the present study, the strength of the age–AMH correlation was assessed both before and after Box–Cox transformation and compared directly with that of the nutritional and iron-status parameters. The comparison was then repeated with age removed from both variables. After first-order partial correlation conditioning on age, every biomarker coefficient attenuated further toward the null, none exceeded 0.10 in absolute value, and the upper confidence limit of the largest estimate was close to the pragmatic 0.200 threshold used to denote limited clinical relevance. The absence of association is therefore not an artefact of the dominant age effect: it survives adjustment for the very factor that might have been expected to mask it.

Vitamin D has emerged as one of the most extensively studied micronutrients in reproductive endocrinology. Experimental studies have identified vitamin D receptors within ovarian tissue, including granulosa cells, suggesting a direct role in folliculogenesis and steroidogenesis [19]. Moreover, vitamin D response elements have been identified in the promoter region of the AMH gene, providing a plausible molecular mechanism through which vitamin D may influence AMH expression [7,12,20]. However, clinical studies evaluating the relationship between serum vitamin D and AMH have yielded conflicting results. Dennis et al. reported a positive association between vitamin D concentrations and AMH levels in late reproductive-aged women [21], whereas other investigations failed to identify a significant relationship after adjustment for age and body mass index [22,23,24,25].

In the present study, serum 25-hydroxyvitamin D did not demonstrate a statistically significant or clinically meaningful association with AMH in either the raw correlation matrix (Pearson’s r = −0.018, *p* = 0.769; Spearman’s rho = 0.063, *p* = 0.309) or the Box–Cox-transformed analysis (Pearson’s r = 0.009, *p* = 0.879; Spearman’s rho = 0.063, *p* = 0.309). This finding is consistent with studies reporting no independent relationship between vitamin D status and AMH after accounting for major determinants such as age and other clinical factors. The discrepancy among studies may be attributable to differences in study populations, ethnic backgrounds, age distributions, vitamin D deficiency prevalence, seasonal variation in vitamin D measurements, and methodological differences in AMH assays. Collectively, current evidence suggests that although vitamin D may influence ovarian physiology, its impact on circulating AMH concentrations remains uncertain and may vary according to specific patient characteristics.

Vitamin B12 and folate are essential cofactors in one-carbon metabolism, DNA synthesis, and methylation reactions, all of which are fundamental for cellular proliferation and reproductive function. Deficiencies in these vitamins have been linked to impaired fertility, adverse pregnancy outcomes, and altered oocyte quality [15]. Nevertheless, relatively few studies have directly investigated their association with AMH. The biological rationale for such an association lies in the importance of methylation pathways for follicular development and granulosa cell function. In our study, neither vitamin B12 nor folic acid demonstrated a significant association with AMH in the raw or Box–Cox-transformed analyses. For vitamin B12, the raw Pearson and Spearman coefficients were −0.027 (*p* = 0.666) and 0.048 (*p* = 0.436), respectively; after Box–Cox transformation, the corresponding values were 0.009 (*p* = 0.887) and 0.048 (*p* = 0.436). Similarly, folic acid showed only weak, non-significant correlations with AMH (raw Pearson’s r = −0.059, *p* = 0.338; Spearman’s rho = −0.039, *p* = 0.533). To our knowledge, evidence regarding the relationship between AMH and vitamin B12 or folate remains limited [15], and our findings therefore contribute valuable information to an area that has received comparatively little scientific attention.

Iron metabolism may also influence female reproductive health through several mechanisms. Iron is essential for cellular respiration, DNA synthesis, and mitochondrial function. Both iron deficiency and iron overload have been implicated in reproductive dysfunction [26]. Ferritin, the primary intracellular iron storage protein, is commonly used as an indicator of iron reserves. Experimental studies suggest that disturbances in iron homeostasis may promote oxidative stress and impair ovarian follicular development [27,28]. Furthermore, severe iron deficiency has been associated with menstrual irregularities and reduced fertility potential.

In the current study, ferritin and other evaluated metabolic parameters did not show clinically meaningful associations with AMH. Ferritin was not significantly correlated with AMH in either the raw analysis (Pearson’s r = 0.118, *p* = 0.055; Spearman’s rho = 0.023, *p* = 0.706) or the Box–Cox-transformed analysis (Pearson’s r = 0.029, *p* = 0.637; Spearman’s rho = 0.023, *p* = 0.706). Similarly, vitamin B12 and folic acid were not significantly associated with AMH. None of the lipid parameters, including triglycerides and total cholesterol, reached statistical significance in either the linear or the monotonic analyses, and all correlation coefficients remained below 0.20, indicating negligible effect sizes and limited clinical relevance. The inconsistency across studies may reflect variations in nutritional status, inflammatory conditions influencing ferritin concentrations, differences in reproductive diagnoses, and the complex bidirectional relationship between iron metabolism and ovarian physiology. Given that ferritin functions not only as an iron-storage marker but also as an acute-phase reactant, interpretation of observed associations should be approached with caution.

An important aspect of the present study is the simultaneous evaluation of multiple nutritional, iron-status and lipid biomarkers in a real-world population undergoing routine reproductive assessment. Unlike studies focusing on a single micronutrient, our approach allows a more comprehensive assessment of factors potentially associated with ovarian reserve. Furthermore, the use of both raw and Box–Cox-transformed bivariate correlation analyses allowed a robust assessment of linear and monotonic relationships, while avoiding unnecessary model complexity given the negligible effect sizes observed for non-age predictors.

Several biological mechanisms may explain potential associations between nutritional status and ovarian reserve [29]. Micronutrients contribute to follicular growth through their roles in antioxidant defense, mitochondrial energy production, DNA repair, epigenetic regulation, and steroid hormone synthesis. Deficiencies may increase oxidative stress within the ovarian microenvironment, leading to accelerated follicular atresia and diminished ovarian function [30]. Conversely, adequate nutritional status may support granulosa cell viability and preserve follicular integrity. Nevertheless, because AMH primarily reflects follicle quantity rather than quality, any effect of micronutrients may be relatively modest compared with the dominant influence of age and genetic determinants. The present study focused on vitamin D, vitamin B12, folate, and ferritin, as these biomarkers are routinely measured in clinical practice and have the most established, though still inconsistent, mechanistic links to AMH transcription and granulosa cell metabolism in the existing literature. Other biomarkers implicated in oxidative stress and follicular health, such as vitamin E, selenium, and direct markers of oxidative stress (e.g., malondialdehyde, total antioxidant capacity), were not available in the present dataset but represent promising candidates for future investigation, given their proposed antioxidant roles in protecting granulosa cells from reactive oxygen species-mediated damage.

From a clinical standpoint, these findings suggest that routine measurement of vitamin D, vitamin B12, folate, or ferritin does not provide meaningful additional information when evaluating ovarian reserve, and that clinical attention should remain focused primarily on chronological age as the dominant determinant of reproductive potential. This conclusion is further reinforced by large-scale, real-world data from assisted reproductive technology (ART) populations. A recent five-year cohort study of over 5000 couples undergoing their first IVF cycle demonstrated that female age remained the most critical determinant of ART outcomes [31]. Similarly, population-based studies examining cumulative live birth rates have consistently shown that female age exerts an overwhelming influence on reproductive success, both directly and through its effect on oocyte yield and quality [32,33]. Taken together with the present findings, this body of evidence supports the view that, in routine clinical practice, resources may be more efficiently directed toward age-based counseling and ovarian reserve testing via AMH or antral follicle count, rather than toward the routine screening of micronutrient or iron-status biomarkers, which appear to offer limited additional predictive value for fertility potential.

The findings of this study should be interpreted in light of several limitations. First, the retrospective cross-sectional design precludes causal inference and limits the ability to determine temporal relationships between nutritional biomarkers and ovarian reserve; because AMH and the biomarkers of interest were measured at a single time point, it is not possible to determine whether biomarker fluctuations precede, follow, or occur independently of changes in ovarian reserve. Prospective longitudinal studies incorporating repeated measurements of AMH alongside serial assessment of vitamin and iron status would allow within-subject changes to be tracked over time and would provide considerably stronger evidence regarding any causal or temporal association between these biomarkers and ovarian reserve decline. Second, information regarding dietary habits, nutritional supplementation, body mass index (BMI), smoking status, physical activity, socioeconomic status, and sunlight exposure was not comprehensively available in the electronic medical records and therefore could not be included in the analysis. These factors could plausibly influence both the biomarker concentrations examined (particularly vitamin D, which is strongly affected by sunlight exposure and BMI) and ovarian reserve itself, and their absence represents a potential source of unmeasured confounding that should be addressed in future prospective studies with more comprehensive data collection. Additionally, vitamin D concentrations were not stratified by season of blood collection, which may have introduced variability given known seasonal fluctuations in vitamin D status. Third, single laboratory measurements may not accurately reflect long-term nutritional status. Fourth, exclusion of women with polycystic ovary syndrome relied on an AMH threshold rather than on a diagnostic assessment, and the limits of that approach are set out under Eligibility Criteria. Women meeting the Rotterdam criteria whose AMH fell below 10 ng/mL will have been retained, and since the syndrome is associated with altered iron and vitamin D status, this constitutes unmeasured confounding of the specific associations examined. Its expected direction, as noted above, is toward creating rather than obscuring an association, so it is unlikely to account for the null results, but it cannot be excluded as a source of residual noise. Additionally, residual confounding from unmeasured reproductive or metabolic variables cannot be excluded. A further point concerns the adjusted analysis: chronological age was the only covariate for which adjustment was possible, and although each first-order partial correlation is equivalent to the age-adjusted coefficient of the corresponding biomarker, a single model entering all nine predictors simultaneously was not fitted, for the reasons of collinearity among the lipid fractions given in the Results. Mutual adjustment among biomarkers is therefore not represented in the reported estimates. Finally, because the study population was derived from a single tertiary-care center, the generalizability of the findings to other populations may be limited. Furthermore, the broad reproductive age range (18–45 years) analyzed in this study, while reflective of the real-world population undergoing routine AMH testing, may have limited the sensitivity to detect associations between AMH and the non-age biomarkers, given the dominant effect of age-related follicular decline across this range; future studies restricting the analysis to a narrower age band may provide a more sensitive assessment of these associations independent of the overwhelming age effect.

Despite these limitations, the study provides clinically relevant information regarding the relationship between AMH and commonly measured biomarkers of vitamin and iron metabolism. The results contribute to the growing body of literature examining potentially modifiable factors associated with ovarian reserve and may assist clinicians in interpreting AMH measurements within a broader metabolic context. Future prospective longitudinal studies incorporating detailed nutritional assessments, dietary interventions, and repeated AMH measurements are warranted to determine whether optimization of micronutrient status can influence ovarian function over time.

## 5. Conclusions

This retrospective study evaluated the associations between serum AMH concentrations and biomarkers of vitamin D status, vitamin B12, folate, ferritin, and lipid profile in 264 women of reproductive age. Age showed a moderate-to-strong inverse association with AMH, both before and after Box–Cox transformation, confirming it as the principal determinant of ovarian reserve. In contrast, none of the investigated nutritional or metabolic biomarkers demonstrated a clinically meaningful association with AMH, with all correlation coefficients remaining below the threshold of practical relevance (r and ρ < 0.20).

These findings indicate that circulating vitamin- and iron-related biomarkers, as well as lipid profile parameters, are unlikely to be materially associated with AMH concentrations beyond the effect of age. Although biological interactions between nutritional status and ovarian function have been proposed, their impact on AMH appears to be minimal compared with age-related ovarian decline. Given the retrospective design and the lack of information on potential confounding factors such as body mass index, dietary habits, and nutritional supplementation, prospective longitudinal studies incorporating repeated AMH measurements are warranted to determine whether these biomarkers have any independent clinical significance in the assessment of ovarian reserve.

Overall, our findings support the view that age remains the most reliable predictor of ovarian reserve, whereas routine measurement of vitamin D, vitamin B12, folate, ferritin, or lipid profile solely for the purpose of interpreting AMH levels is unlikely to provide additional clinical value.

## Figures and Tables

**Table 5:** Distribution of vitamin D status in the study population according to conventional clinical thresholds (n = 264).

Vitamin D Status	n	%
Deficiency (<20 ng/mL)	113	42.8
Insufficiency (20–29.9 ng/mL)	90	34.1
Sufficiency (≥30 ng/mL)	61	23.1

## Data Availability

Authors can confirm that all relevant data are included in the article and/or Supplementary Materials.

## Supplementary Materials

The following supporting information can be downloaded at: https://www.mdpi.com/article/10.3390/jcm15197571/s1, Table S1. Baseline Distributional Characteristics and Estimated Box–Cox Transformation Parameters (λ) with 95% Confidence Intervals for Continuous Study Variables (n = 264); Table S2. Sensitivity power analysis across hypothesized partial correlation coefficients for an age-adjusted model (n = 264, α = 0.05, two-sided).
