# Supplementary material for: Routine Vitamin and Iron Biomarkers Do Not Explain Variation in Anti-Müllerian Hormone: Implications for Ovarian Reserve Testing in Clinical Practice
Source: J Clin Med. 2026 Sep 29;15(19):7571. doi: 10.3390/jcm15197571 (PMC13666060; doi:10.3390/jcm15197571)
Supplement: Supplementary file 1 [file jcm-15-07571-s001.zip › jcm-4550225-supplementary.pdf]

Supplementary Table S1. Baseline Distributional Characteristics and Estimated Box-Cox Transformation Parameters (λ) with 95% Confidence Intervals for Continuous Study Variables (n = 264)

Variable	Raw Mean ± SD	Skewness (Raw)	Optimal λ Estimate	95% Confidence Interval for λ	Transformed Name
**AMH**	1.58 ± 1.71	1.699	0.262	(0.187, 0.338)	AMH_bc
**TG**	93.99 ± 78.61	4.245	-0.499	(-0.685, -0.320)	TG_bc
**HDL_C**	57.90 ± 13.83	0.41	0.413	(-0.001, 0.828)	HDL_C_bc
**LDL_C**	112.38 ± 27.64	0.216	0.542	(0.107, 0.985)	LDL_C_bc
**TC**	189.03 ± 35.58	0.628	-0.085	(-0.618, 0.446)	TC_bc
**Ferritin**	36.24 ± 43.98	3.531	0.003	(-0.097, 0.102)	Ferritin_bc
**Vit_B12**	371.97 ± 195.53	2.9	-0.263	(-0.476, -0.054)	Vit_B12_bc
**Folic_Acid**	7.40 ± 3.54	1.808	0.04	(-0.178, 0.256)	Folic_Acid_bc
**Vit_D_25OH**	23.22 ± 14.88	2.846	0.169	(0.019, 0.318)	Vit_D_25OH_bc

Supplementary Table S2. Sensitivity power analysis across hypothesized partial correlation coefficients for an age-adjusted model (n = 264, $\alpha = 0.05$, two-sided).

Hypothes□zed part□al r	Cohen f^2	n	Covar□ates (k)	Power (1 − β)
**0.100**	0.010	264	1	0.369
**0.150**	0.023	264	1	0.688
**0.172**	0.030	264	1	0.803
**0.200**	0.042	264	1	0.910
**0.250**	0.067	264	1	0.987
**0.300**	0.099	264	1	0.999

Note: Cohen's f^2 was computed as $r^2/(1 - r^2)$. Power was derived from the non-central F distribution for the general linear model with one conditioning covariate (chronological age), at a two-sided significance threshold of $\alpha = 0.05$.
